# Clinically feasible automated MRI volumetry of the brain as a prognostic marker in subjective and mild cognitive impairment

**DOI:** 10.3389/fneur.2024.1425502

**Published:** 2024-07-01

**Authors:** Rachel Amland, Geir Selbæk, Anne Brækhus, Trine H. Edwin, Knut Engedal, Anne-Brita Knapskog, Ellen Regine Olsrud, Karin Persson

**Affiliations:** ^1^The Norwegian National Centre for Ageing and Health, Vestfold Hospital Trust, Tønsberg, Norway; ^2^Department of Geriatric Medicine, Oslo University Hospital, Oslo, Norway; ^3^Faculty of Medicine, Institute of Health and Society, University of Oslo, Oslo, Norway; ^4^Faculty of Medicine, Institute of Clinical Medicine, University of Oslo, Oslo, Norway; ^5^Department of Neurology, Oslo University Hospital, Oslo, Norway; ^6^Department of Radiography Ullevål, Division of Radiology and Nuclear Medicine, Oslo University Hospital, Oslo, Norway

**Keywords:** subjective cognitive decline, mild cognitive impairment, magnetic resonance imaging, automated volumetry NeuroQuant®, dementia, disease progression, Alzheimer’s disease, Clinical Dementia Rating Scale

## Abstract

**Background/aims:**

The number of patients suffering from cognitive decline and dementia increases, and new possible treatments are being developed. Thus, the need for time efficient and cost-effective methods to facilitate an early diagnosis and prediction of future cognitive decline in patients with early cognitive symptoms is becoming increasingly important. The aim of this study was to evaluate whether an MRI based software, NeuroQuant® (NQ), producing volumetry of the hippocampus and whole brain volume (WBV) could predict: (1) conversion from subjective cognitive decline (SCD) at baseline to mild cognitive impairment (MCI) or dementia at follow-up, and from MCI at baseline to dementia at follow-up and (2) progression of cognitive and functional decline defined as an annual increase in the Clinical Dementia Rating Scale Sum of Boxes (CDR-SB) score.

**Methods:**

MRI was performed in 156 patients with SCD or MCI from the memory clinic at Oslo University Hospital (OUH) that had been assessed with NQ and had a clinical follow-up examination. Logistic and linear regression analyses were performed with hippocampus volume and WBV as independent variables, and conversion or progression as dependent variables, adjusting for demographic and other relevant covariates including Mini-Mental State Examination-Norwegian Revised Version score (MMSE-NR) and Apolipoprotein E ɛ4 (*APOE* ɛ4) carrier status.

**Results:**

Hippocampus volume, but not WBV, was associated with conversion to MCI or dementia, but neither were associated with conversion when adjusting for MMSE-NR. Both hippocampus volume and WBV were associated with progression as measured by the annual change in CDR-SB score in both unadjusted and adjusted analyses.

**Conclusion:**

The results indicate that automated regional MRI volumetry of the hippocampus and WBV can be useful in predicting further cognitive decline in patients with early cognitive symptoms.

## Introduction

According to current estimates, approximately 55 million people worldwide are suffering from dementia, and the number is expected to nearly triple by 2050 (1). The knowledge on dementia prevention has increased and several treatment trials targeting early stages of the various etiological causes of dementia, e.g., Alzheimer’s disease (AD) and Lewy body disease, are ongoing (2–5). To utilize this new knowledge the need for time efficient and cost-effective methods to facilitate early diagnosis and prediction of future cognitive decline is becoming increasingly important.

Studies have shown that, among patients with MCI, approximately 15% develop dementia after two years; however, some patients never convert to dementia whereas some revert to normal functioning (6, 7). Moreover, the risk of progressing from SCD and MCI to dementia has been found to vary according to the population being studied, e.g., a higher conversion rate has been found in patients in specialized memory clinics than among their counterparts in community-based cohorts (8, 9). Factors such as the degree of memory impairment, altered levels of amyloid-β and tau-proteins in the cerebral fluid, and abnormal fluorodeoxyglucose (FDG) positron emission tomography (PET) together with *APOE* ɛ4 genotype and atrophy detected on magnetic resonance imaging (MRI), have been found to predict conversion from MCI to dementia (10, 11). Also, several other MRI based methods are available in research, but not yet clinically available (12, 13). Amyloid PET is regarded a valuable diagnostic marker of AD but has not been found to have a high accuracy in predicting conversion from MCI to AD (14). The estimation of medial temporal lobe atrophy (MTA) on MRI scans is considered a useful predictor of conversion from MCI to AD dementia (15). Similarly, hippocampus atrophy has been linked to AD conversion and progression (16, 17). Likewise, lower whole brain volume (WBV) has been linked to both worse global cognition over time and conversion from MCI to AD (18, 19). However, less is known about the use of structural MRI of the brain as a prognostic factor in SCD, and studies have shown contradictory results in recent years when comparing patients with SCD with healthy controls (20, 21). Cross-sectional studies conducted in patients with SCD in memory clinic settings have demonstrated a small reduction in brain volume within regions associated with early-stage AD when compared to healthy controls (22–24). These findings suggest potential for detection of early changes during the preclinical stage. Conversely, other studies have reported no significant differences in hippocampal volume between patients with SCD and healthy controls (25, 26).

Visual evaluation and automatic volumetry are two ways of assessing structural changes in the brain based on MRI. When used to evaluate the hippocampal region, both methods have been found to be equally good at differentiating patients with AD dementia from patients without dementia in a previous study from the memory clinic at Oslo University Hospital (OUH) (27). Further, in a review from 2021, Pini et al. (28) suggest that automatic methods appear to be more sensitive than manual methods to identify preclinical neurodegenerative changes. NeuroQuant® (NQ) is a clinically feasible software that automatically produces volumetric data without the expertise of a neuroradiologist. It was taken into use at the memory clinic at OUH in 2009 as it was the first software approved for clinical use by the US Food and Drug Administration (FDA) and being CE marked. It has been validated against FreeSurfer, a widely used semi-automated volumetry method, previously but as opposed to FreeSurfer it is automatic and thus clinically feasible (29). A recent study from Persson et al. (30) comparing NQ volumetry to visual evaluation showed a high correlation between the two methods, and NQ volumetry of the hippocampi and temporal regions to be substantially better in discriminating dementia from non-dementia (compared to the visual MTA-scale). However, studies evaluating the inter-method reliability between different available volumetry software programs have found varying results from good agreement to lack of interchangeability between the programs, which is important to take into account in clinical settings (31, 32). In comparison to other neuroimaging modalities, MRI is widely available and the cost is modest compared to for instance PET-scans (33). Taking these aspects in consideration automatic volumetry may have particular relevance in a primary care setting and locations lacking neuroradiologists with the necessary experience in visual evaluation, as a less costly and feasible diagnostic method in clinical settings.

In the present prospective cohort study from the memory clinic at OUH, two NQ measures, i.e., the AD associated hippocampus volume, and the more general WBV, were used to detect early neurodegenerative changes. The aims were to evaluate whether these volumetry measures could be used to (1) predict progression to a more impaired level of cognitive functioning—that is, conversion from SCD at baseline to MCI or dementia at follow-up, and from MCI at baseline to dementia at follow-up, and (2) to predict progression of cognitive and functional impairment defined as an annual increase in the Clinical Dementia Rating Scale Sum of Boxes (CDR-SB) score.

## Materials and methods

### Participants

**Figure 1:**
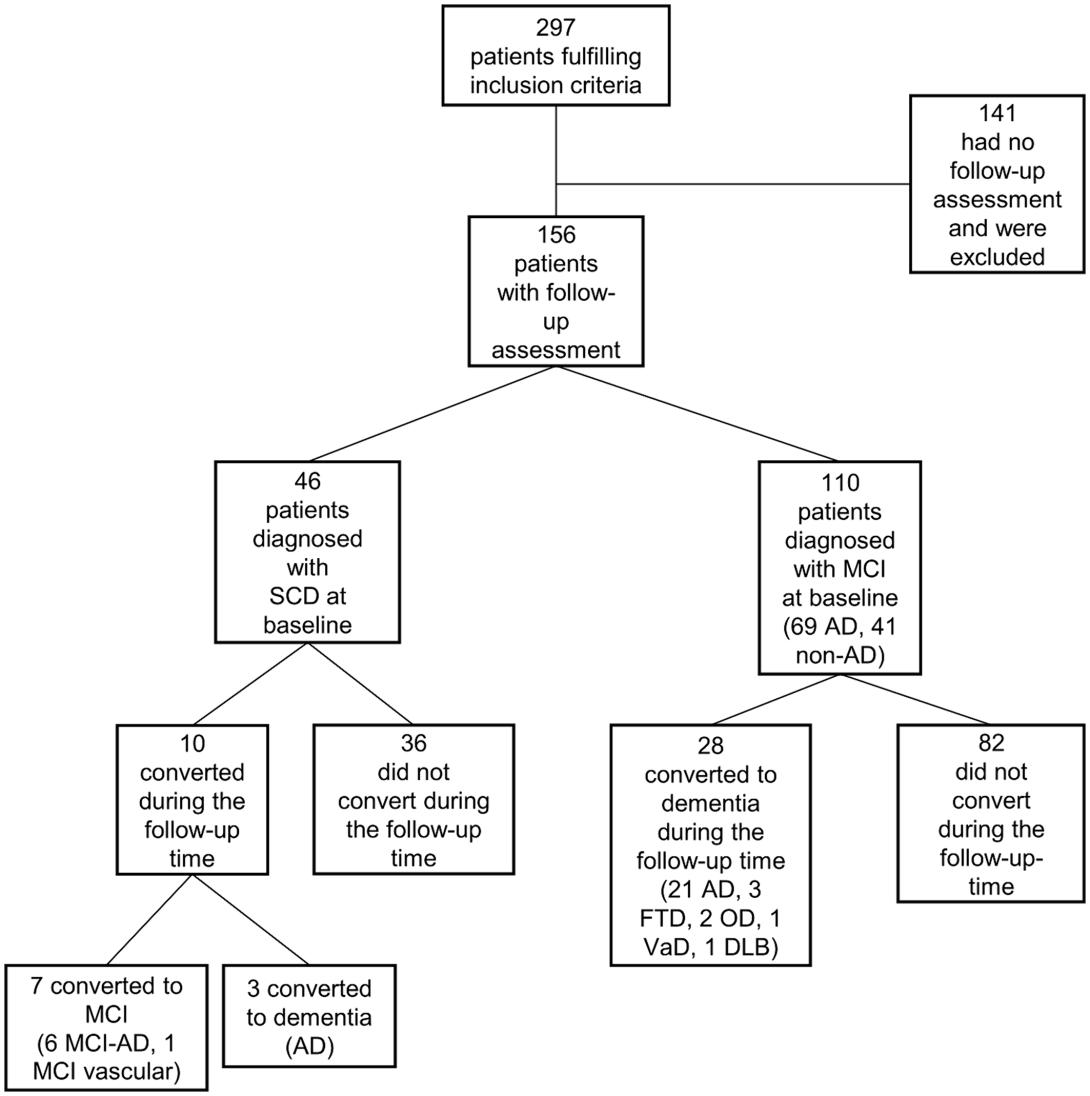
Flow chart. SCD, subjective cognitive decline; MCI, mild cognitive impairment, AD, Alzheimer’s disease; DLB, dementia with Lewy bodies; FTD, frontotemporal dementia; OD, other dementia; VaD, vascular dementia.

A total of 297 patients examined at the memory clinic at OUH between 2010 and 2020, diagnosed with SCD (*n* = 99) or MCI (*n* = 198), who had been examined with MRI of the brain including assessment with NQ, were eligible for inclusion. There was no systematic selection for referral to NQ MRI. All patients had given a written consent to be included in the Norwegian Registry of Persons Assessed for Cognitive Symptoms (NorCog) (34) which is a national quality and research registry. In NorCog, data is collected from a standardized assessment at hospital outpatient clinics in Norway. By the end of 2022, 44 hospitals participated in collecting data. The acceptance rate of inclusion in NorCog is above 90% at the memory clinic at OUH. Of the 297 patients, 156 (46 diagnosed with SCD and 110 diagnosed with MCI at baseline) had at least one clinical follow-up examination and were included in this prospective longitudinal study. If a patient had multiple follow-up consultations the last visit was registered and utilized to evaluate longitudinal progression. The mean follow-up time was 32.6 months (range: 7–91 months). Overall, 141 patients, were not followed up, possibly due to a lack of clinical indication for additional evaluation or because patients declined any follow-up consultations (see Figure 1 for details). Analyses comparing patients who were followed up (*n =* 156) with those who were not (*n =* 141) showed no significant difference in age (*p* 0.398), sex (*p* 0.688), education (*p* 0.271), CDR-SB scores (*p* 0.196) or the Mini-Mental State Examination-Norwegian Revision (MMSE-NR) scores (*p* 0.055).

### Clinical assessments

All patients were examined using a standardized protocol as part of the NorCog registry (34). Data from the thorough cognitive test battery as well as information about demography, symptoms, information from a next-of-kin, and the clinical diagnosis is included in the NorCog database. Patients are assessed with computed tomography (CT) or MRI of the brain as part of the clinical diagnostic work-up. According to clinical indication, further examinations of cerebrospinal fluid and advanced imaging are performed.

In the present study, the MMSE-NR, with scores ranging from 0 to 30 and with higher values indicating better cognitive functioning was used as a measure of cognitive function at baseline (35).

The CDR was used as a measure of the level of cognitive and functional impairment. It rates a person’s abilities in six cognitive and functional domains (i.e., memory, orientation, judgment and problem-solving, community affairs, home and hobbies, and personal care) scored 0, 0.5, 1, 2, or 3, wherein a higher score indicates more severe impairment (36). The CDR was scored by one of three CDR-certified researchers (i.e., T.H.E., K.P., or R.A.) based on the available information from the patients’ records, including information from an interview with the caregiver. The CDR was scored by the same researcher at baseline and follow-up. In research, the scores from the six CDR items are often summed up to form a continuous scale, the CDR sum of boxes (CDR-SB) with scores ranging from 0 to 18 and with higher scores indicating more severe impairment (37). In the present study, the annual change in CDR-SB was calculated and used as a measure of disease progression [(CDR-SB at follow-up – CDR-SB baseline)/months of follow-up time *12]. In 25 patients, the annual change in CDR-SB could not be calculated retrospectively due to insufficient information in the patient records at baseline or follow-up to assess CDR.

### Diagnoses

Diagnoses were made retrospectively based on all available information from the examinations at baseline and at follow-up. Diagnoses were made by one of three researchers who are also experienced clinicians (T.H.E, K.P, or R.A). In inconclusive cases, two experienced senior clinicians were consulted (A.B.K, G.S). Diagnoses of SCD were made based on the Jessen criteria (38), The National Institute on Aging and Alzheimer’s Association (NIA-AA) diagnostic criteria were used for the diagnosis of MCI (39), and both the Diagnostic and Statistical Manual of Mental Disorders, Fifth Edition, (DSM-5) diagnostic criteria (40) and NIA-AA criteria were used for the diagnosis of dementia (41). At baseline the MCI patients were diagnosed with AD or non-AD etiology according to the clinical NIA-AA diagnostic criteria for AD (39, 41). Converters were defined as patients who progressed to a more impaired level of cognitive functioning—that is, from SCD at baseline to MCI or dementia at follow-up, or from MCI at baseline to dementia at follow-up. Further, at follow-up, all converters were diagnosed etiologically according to the clinical NIA-AA diagnostic criteria for AD (AD-MCI or AD-dementia) (39, 41); the Vascular Behavioral and Cognitive Disorders (VASCOG) criteria for vascular cognitive impairment (42); the 2017 McKeith criteria for the diagnosis of dementia with Lewy bodies (43); and the Rascovsky and Gorno-Tempini criteria for the behavior and primary progressive aphasia variants of frontotemporal dementia (44, 45). If none of these were present, the etiology was denoted dementia due to other etiology (OD). Converters with AD mixed with vascular etiologies or other neurodegenerative diseases were regarded as having AD. See Figure 1 for details. Specific AD biomarkers from cerebrospinal fluid or amyloid positron emission tomography were not included in the diagnostic evaluation as these examinations were only performed on clinical indication and were only available in 64 of the included patients.

### MRI assessments

**Figure 2:**
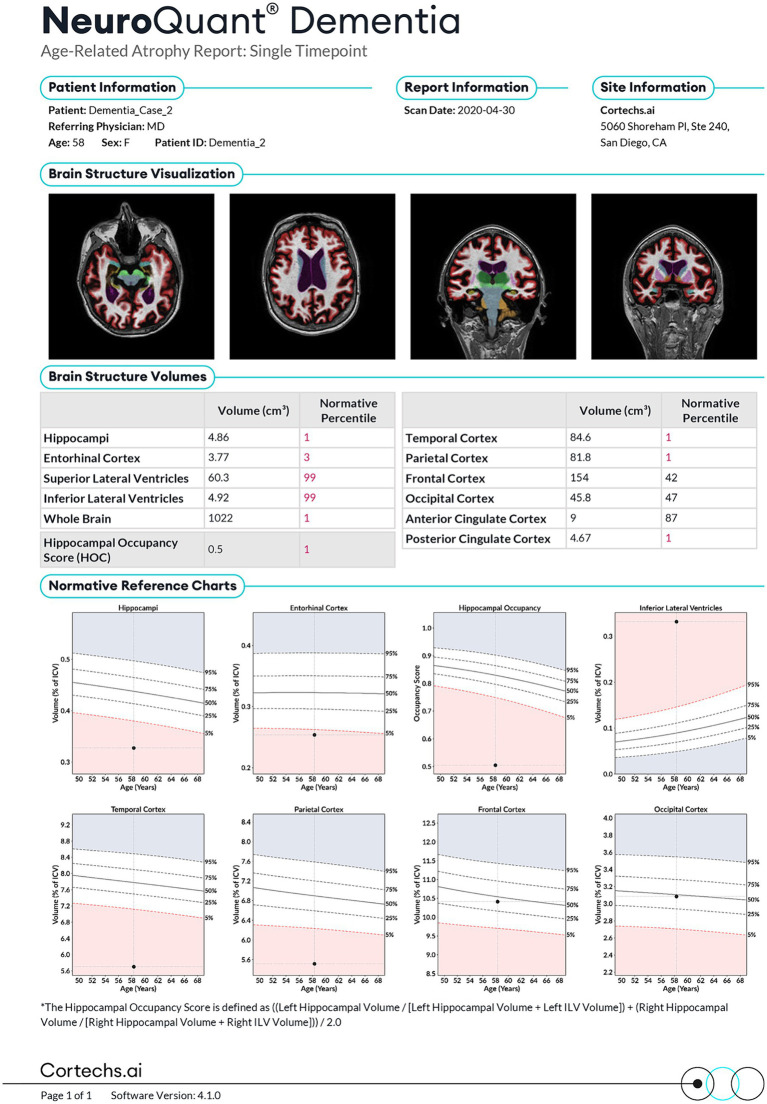
The NeuroQuant® (NQ) report. Published with permission from Corthex.ai.

T1-weighted 3D scanning was performed on a 3 Tesla MRI scanner (GE Signa HDxt) that was upgraded to GE Discovery MR750 in 2015. All of the included patients were scanned with a study specific protocol, including similar 3D T1-w gradient echo sequence. The scans were assessed with the clinically Food and Drug Administration (FDA)-approved NeuroQuant® software, versions 1, 2, and 3 (NQ, CorTechs Labs–University of California, San Diego, CA, United States). NQ performs automatic segmentation and provides volumes of several brain structures as well as volumes expressed as percentages of total intracranial volume (ICV) to adjust for head size. Along with those volumes, the volumes for some structures are compared with a normative data set, and an age- and sex-adjusted percentile is reported (Figure 2). In our study, two measures were included in the analyses. First, the clinically applicable hippocampus percentile because hippocampus atrophy is known to be strongly associated with AD (16, 17). All hippocampus percentile calculations were based on NQ version 1 and 2. Secondly, as we wanted to evaluate the ability of MRI-NQ to predict progression in a heterogeneous SCD and MCI population and not only AD, we included WBV as a second predictor. A lower cerebral volume has been found to be associated with faster cognitive decline in memory and global cognition, in several neurodegenerative pathologies (18, 46, 47). WBV was only available as a percentage of ICV, not as a percentile. All whole brain calculations were performed using NQ version 3. As stated, only patients who had been referred to an MRI of the brain including an assessment using NQ were included in the study. The only reason for not referring a patient to this assessment was if MRI was contraindicated or if the patient had already been examined with MRI previous to the referral to the memory clinic (this was the case in 80% of the patients being examined at the memory clinic).

### Other assessments

*APOE* ɛ4 genotype is associated with cognitive decline and atrophy in medial temporal regions and was therefore included as a covariate (48, 49). *APOE* genotyping was performed in 132 patients (37 diagnosed with SCD at baseline and 95 diagnosed with MCI at baseline) at deCODE Genetics (Reykjavik, Iceland) using the Illumina Infinium OmniExpress v1.1 chip. The results were dichotomized according to *APOE* ɛ4 status (i.e., 0 for no *APOE* ɛ4 allele and 1 if at least one allele of *APOE* ɛ4 was present).

### Statistical analyses

The de-identified data were analyzed using IBM SPSS Statistics for Windows version 28 (Armonk, NY, United States) To compare demographic and clinical characteristics between groups, the chi-squared test was used for categorical variables and the independent sample *t* test for continuous variables. The significance level was set at *p* < 0.05.

Bivariate correlation analyses were performed between the independent variables in advance of the regression analyses to build the adjusted models and to avoid situations introducing multicollinearity. Age, sex, and MMSE-NR were included as covariates in all models while education, follow-up time, MRI scanner, and NQ version were only adjusted for if the association to the dependent variable was *p* < 0.2. Adjusted regression analyses with all covariates included are available in the Supplementary Tables S1, S2. First, logistic regression was used to explore the predictive properties of hippocampus percentile and WBV on conversion to MCI or dementia. On that count, two separate analyses were conducted. In the first, exploring the predictive value of hippocampus percentile, two models were created; in model 1, we adjusted for age, sex, and follow-up time, and in model 2, we additionally adjusted for MMSE-NR score at baseline to adjust for cognitive stage. In the second logistic regression analysis the same steps as for hippocampus percentile were taken to explore the predictive value of WBV. Additionally logistic regression analyses were conducted to explore the predictive properties of the NQ volumetrics on conversion from SCD or MCI to AD-MCI or AD-dementia, adjusting for age, sex, follow-up time and MMSE-NR. Secondly, multiple linear regression analyses were performed to explore the predictive properties of the hippocampus percentile and WBV on the progression of cognitive and functional decline, measured as the annual change in CDR-SB score. Again, we explored these predictive properties in separate analyses, one on the hippocampus percentile and one on WBV as predictors of progression. And again, model 1 adjusted for age, sex, and follow-up time, and MMSE-NR at baseline was added in model 2.

Only patients with complete data were included in the regression analyses, i.e., 141 in the logistic regression models and 117 in the linear regression models. Lastly, in a subgroup of patients with available *APOE* ɛ4 carrier status, we repeated the analyses on conversion (*n =* 120) and progression (*n =* 100) by adjusting for the same variables as in the previous regression analyses with the addition of *APOE* ɛ4 carrier status. In this cohort with available *APOE* status, education was found to have a *p* value below 0.2 in the unadjusted analyses and was therefore included as a covariate in these models. Stratified analyses based on *APOE* ɛ4- carrier status were carried out to explore potential differences in the predictive value of the MRI measures on conversion between the *APOE* ɛ4 -positive group and the *APOE* ɛ4-negative group.

## Results

Table 1 shows patient characteristics according to whether they were converters or non-converters. Of the 38 converters 7 converted from SCD to MCI, 3 from SCD to dementia, and 28 converted from MCI to dementia during the follow-up-time (Figure 1). The converters had a significantly higher age, greater annual change in CDR-SB score, and longer follow-up time, as well as significantly lower hippocampus percentile and WBV, than non-converters. Among the converters, 63% were *APOE* ɛ4 carriers, compared with 41% in the non-converter group. Figure 1 includes baseline diagnoses (AD, non-AD) and the etiological diagnoses of the converters at follow up. Characteristics of the patients who converted and those who did not convert, limited to the SCD patient group (*n* = 46) are available in the Supplementary Table S3.

**Table 1:** Characteristics of patients.

	Converters *n* 38	Non-converters *n* 118	*p*ᶧ
Age, years	69.6 (7.2)	63.4 (10.2)	**0.001**
Female, *n* (%)	21 (55.3)	49 (41.5)	0.139
Education, years	13.7 (3.6)	14.3 (3.4)	0.393
MMSE-NR score at baseline	27.4 (2.5)	28.2 (2.0)	0.091
CDR-SB score at baseline	1.19 (1.0)	0.98 (0.8)	0.256
Annual change in CDR-SB score*	1.14 (0.7)	−0.05 (0.5)	**<0.001**
APOE ɛ4 carriers, *n* (%)**	20 (62.5)	41 (41.0)	**0.034**
Hippocampus percentile	35.3 (31.2)	53.7 (32.3)	**0.002**
Whole brain volume	72.7 (3.4)	74.4 (3.9)	**0.012**
Follow-up time (in years)	3.2 (1.5)	2.6 (1.4)	**0.029**

All continuous variables are expressed as mean (SD). Bold values highlight significant differences (*p* < 0.05 two-tailed); MMSE-NR: Mini-Mental State Examination-Norwegian revision; CDR-SB: Clinical Dementia Rating Scale Sum of Boxes; ᶧStudent’s t test/χ2 test. *n 131, ** *n* 132.

The logistic regression analysis revealed a significant association between hippocampus percentile and conversion in the unadjusted analyses (OR 0.98, *p* 0.002), as well as in the adjusted model 1 (OR 0.98, *p* 0.028). In model 2, which included MMSE-NR as a covariate, the association was no longer significant (OR 0.99, *p* 0.060), see Table 2. The association between WBV and conversion was significant in the unadjusted analysis (OR 0.89, *p* 0.022), but was no longer significant in the adjusted models 1 or 2 (OR 0.91, *p* 0.188 and OR 0.91, *p* 0.907, respectively), see Table 3. Sub-analyses on conversion to AD-MCI and AD-dementia did not differ substantially from the whole group analyses (hippocampus percentile OR 0.98, *p* 0,069; WBV OR 0.92, *p* 0.322, both adjusted models 2, Supplementary Table S4).

**Table 2:** Logistic regression models with converters (1) and non-converters (0) as the dependent variable involving 141 patients with complete data.

	Unadjusted model		Adjusted model 1		Adjusted model 2	
	OR (95% CI)	*p*	OR (95% CI)	*p*	OR (95% CI)	*p*
Age	1.08 (1.03–1.13)	**0.002**	1.05 (1.00–1.11)	0.064	1.05 (0.99–1.10)	0.080
Sex	0.67 (0.31–1.14)	0.304	0.53 (0.22–1.24)	0.141	0.52 (0.22–1.24)	0.142
Education	0.95 (0.85–1.06)	0.360				
Hippocampus percentile	0.98 (0.97–0.99)	**0.002**	0.98 (0.97–1.00)	**0.028**	0.99 (0.97–1.00)	0.060
Follow-up time	1.03 (1.01–1.05)	**0.014**	1.03 (1.01–1.05)	**0.015**	1.04 (1.01–1.06)	**0.002**
MRI scanner before 2015	1.12 (0.55–2.53)	0.669				
NQ version 1 or 2	1.07 (0.32–3.59)	0.915				
MMSE-NR score	0.85 (0.72–1.01)	0.063			0.87 (0.72–1.05)	0.138
*R* ^2^			0.22		0.24	

Hippocampus percentile as the independent variable.

Values in bold are significant, OR: odds ratio; CI: confidence interval; *R*^2^: explained variance, NQ: NeuroQuant®; MMSE-NR: Mini-Mental State Examination-Norwegian revision.

**Table 3:** Logistic regression models with converters (1) and non-converters (0) as the dependent variable involving 141 patients with complete data.

	Unadjusted		Adjusted model 1		Adjusted model 2	
	OR (95% CI)	*p*	OR (95% CI)	*p*	OR (95% CI)	*p*
Age	1.08 (1.03–1.13)	**0.002**	1.05 (0.99–1.11)	0.146	1.04 (0.98–1.10)	0.231
Sex	0.67 (0.31–1.14)	0.304	0.60 (0.26–1.38)	0.229	0.57 (0.25–1.33)	0.192
Education	0.95 (0.85–1.06)	0.360				
Whole brain volume	0.89 (0.80–0.98)	**0.022**	0.91 (0.79–1.05)	0.188	0.91 (0.78–1.05)	0.907
Follow-up time	1.03 (1.01–1.05)	**0.014**	1.04 (1.01–1.06)	**0.006**	1.04 (1.01–1.07)	**0.002**
MRI scanner before 2015	1.12 (0.55–2.53)	0.669				
MMSE-NR score	0.85 (0.72–1.01)	0.063			0.84 (0.70–1.01)	0.057
*R* ^2^			0.19		0.22	

Whole brain volume as the independent variable.

Values in bold are significant, OR: odds ratio; CI: confidence interval; *R*^2^: explained variance; NQ: NeuroQuant®; MMSE-NR: Mini-Mental State Examination-Norwegian revision.

Multiple linear regression analyses showed that both hippocampus percentile and WBV were significantly associated with annual change in CDR-SB score in both unadjusted and adjusted analyses (model 2 of each volumetric measure −0.23, *p* 0.019 (hippocampus percentile) vs. -0.28 *p* 0.013 (WBV)), see Tables 4, 5.

**Table 4:** Linear regression analyses of annual change in CDR-SB scores involving 117 patients with complete data.

	Unadjusted model		Adjusted model 1		Adjusted model 2	
	*β* (*SE*)	*p*	*β* (*SE*)	*p*	*β* (*SE*)	*p*
Age	0.31 (0.001)	**<0.001**	0.18 (0.001)	0.057	0.17 (0.001)	0.057
Sex	−0.56 (0.012)	0.551	−0.11 (0.011)	0.189	−0.10 (0.011)	0.218
Education	−0.11 (0.002)	0.244				
Hippocampus percentile	−0.38 (<0.001)	**<0.001**	−0.30 (<0.001)	**0.002**	−0.23 (<0.001)	**0.019**
MMSE-NR score	−0.25 (0.003)	**0.008**			−0.19 (0.003)	**0.032**
Follow-up time	0.22 (<0.001)	**0.019**	0.18 (<0.001)	**0.035**	0.17 (<0.001)	**0.046**
MRI scanner before 2015	0.95 (0.012)	0.307				
NQ version 1 or 2	0.05 (0.020)	0.594				
*R* ^2^			0.14		0.24	

Hippocampus percentile as the independent variable.

Values in bold are significant. *β*: coefficient; SE: Standard error; *R*^2^: explained variance; CDR − SB: Clinical Dementia Rating Scale Sum of Boxes; NQ: NeuroQuant®; MMSE: Mini-Mental State Examination-Norwegian revision.

**Table 5:** Linear regression analyses of annual change in CDR-SB scores involving 117 patients with complete data.

	Unadjusted		Adjusted model 1		Adjusted model 2	
	β (*SE*)	*p*	*β* (*SE*)	*p*	*β* (SE)	*p*
Age	0.31 (0.001)	**<0.001**	0.14 (0.001)	0.224	0.09 (0.001)	0.423
Sex	−0.56 (0.012)	0.551	−0.07 (0.011)	0.431	−0.82 (0.011)	0.412
Education	−0.11 (0.002)	0.244				
Whole brain volume	−0.28 (0.002)	**0.003**	−0.27 (0.002)	**0.022**	−0.28 (0.002)	**0.013**
MMSE-NR score	−0.25 (0.003)	**0.008**			−0.27 (0.003)	**0.002**
Follow-up time	0.22 (<0.001)	**0.019**	0.29 (<0.001)	**0.002**	0.35 (<0.001)	**<0.001**
MRI scanner before 2015	0.95 (0.012)	0.307				
*R* ^2^			0.182		0.249	

Whole brain volume as the independent variable.

Values in bold are significant. *β*: coefficient; SE: Standard error; *R*^2^: explained variance; CDR-SB: Clinical Dementia Rating Scale Sum of Boxes; NQ: NeuroQuant®; MMSE-NR: Mini-Mental State Examination-Norwegian revision.

Analyses in the subsample with available *APOE* ɛ4 results (*n* = 120), including *APOE* ɛ4 status as a covariate, showed an association between hippocampus percentile and conversion in both models 1 and 2 (i.e., an association with conversion was found in model 2 only when *APOE* ɛ4 was included). The association of WBV and conversion did not change when *APOE* ɛ4 status was added to the models. When stratified by *APOE* ɛ4 status, the hippocampus percentile was significantly associated with conversion in the *APOE* ɛ4 -negative group (OR 0.96, *p* 0.035) but not in the *APOE* ɛ4 -positive group (OR 0.99, *p* 0 0.184). This difference was not found for WBV (*APOE* ɛ4 -negative group: OR 0.92, *p* 0.567; *APOE* ɛ4 -positive group OR 0.95, *p* 0.618). Both MRI measures remained associated with progression (annual change CDR-SB score) when *APOE* ɛ4 was added as a covariate (Tables 6–9).

**Table 6:** Logistic regression with converters (1) and non-converters (0) as the dependent variable involving 120 patients with complete data, including APOE **ɛ**4-carriers status.

	Unadjusted model		Adjusted model 1		Adjusted model 2	
	OR (95% CI)	*p*	OR (95% CI)	*p*	OR (95%CI)	*p*
Age	1.09 (1.03–1.14)	**0.002**	1.05 (0.99–1.11)	0.112	1.05 (0.99–1.11)	0.116
Sex	0.70 (0.30–1.60)	0.431	0.51 (0.19–1.38)	0.182	0.52 (0.19–1.41)	0.196
Education	0.94 (0.83–1.06)	0.296				
Hippocampus percentile	0.98 (0.96–0.99)	**0.001**	0.98 (0.96–1.00)	**0.013**	0.98 (0.96–1.00)	**0.025**
Follow-up time	1.03 (1.00–1.05)	**0.032**	1.03 (1.00–1.06)	**0.043**	1.03 (1.00–1.06)	**0.032**
MRI NQ before 2015	1.09 (0.48–2.52)	0.832				
MR version 1 or 2	0.96 (0,40–2.22)	0.915				
MMSE-NR score	0.86 (0.73–1.03)	0.097			0.92 (0.76–1.12)	0.395
APOE ε4-carrier	2.59 (1.10–6.09)	**0.029**	1.44 (0.54–3.83)	0.469	1.34 (0.49–3.66)	0.595
*R* ^2^			0.27		0.28	

Hippocampus percentile as the independent variable.

Values in bold are significant, OR: odds ratio; CI: confidence interval; *R*^2^: explained variance; NQ: NeuroQuant®; MMSE-NR: Mini-Mental State Examination-Norwegian revision.

**Table 7:** Logistic regression with converters (1) and non-converters (0) as the dependent variable involving 120 patients with complete data including APOE **ɛ**4-carrier status.

	Unadjusted model		Adjusted model 1		Adjusted model 2	
	OR (95% CI)	*p*	OR (95% CI)	*p*	OR (95%CI)	*p*
Age	1.08 (1.03–1.12)	**0.001**	1.06 (0.99–1.13)	0.125	1.05 (0.98–1.13)	0.162
Sex	0.72 (0.31–1.64)	0.431	0.68 (0.27–1.74)	0.421	0.66 (0.26–1.71)	0.394
Education	0.93 (0.83–1.06)	0.276				
Whole brain volume	0.88 (0.79–0.99)	**0.026**	0.93 (0.79–1.08)	0.336	0.93 (0.79–1.09)	0.349
Follow-up time	1.03 (1.00–1.05)	**0.029**	1.03 (1.00–1.06)	**0.033**	1.04 (1.01–1.07)	**0.019**
MRI NQ before 2015	1.12 (0.49–2.57)	0.788				
MMSE-NR score	0.86 (0.73–1.02)	0.091			0.87 (0.72–1.05)	0.149
APOE ε4-carrier	2.64 (1.12–6.19)	**0.026**	1.48 (0.57–3.85)	0.423	1.36 (0.51–3.61)	0.538
*R* ^2^			0.21		0.23	

Whole brain volume as the independent variable.

Values in bold are significant, OR: odds ratio; CI: confidence interval; *R*^2^: explained variance; NQ: NeuroQuant®; MMSE-NR: Mini-Mental State Examination-Norwegian revision.

**Table 8:** Linear regression analysis of annual change in CDR-SB scores involving 100 patients with complete data, including APOE **ɛ**4-carrier status.

	Unadjusted model		Adjusted Model 1		Adjusted model 2	
	*β* (SE)	*p*	*β* (*SE*)	*p*	*β* (*SE*)	*p*
Age	0.33 (0.001)	**0.004**	0.15 (0.001)	0.148	0.16 (0.001)	0.119
Sex	−0.42 (0.013)	0.677	−0.11 (0.011)	0.235	−0.08 (0.011)	0.328
Education	−0.16 (0.002)	0.119			−0.76 (0.002)	0.408
Hippocampus percentile	−0.45 (<0.001)	**<0.001**	−0.38 (<0.001)	**<0.001**	−0.33 (0.000)	**0.003**
MMSE-NR score	−0.25 (0.003)	**0.011**			−0.17 (0.003)	0.084
Follow-up time	0.26 (0.000)	**0.011**	0.22 (<0.001)	**0.017**	0.26 (0.00)	**0.007**
MRI NQ before 2015	0.12 (0.013)	0.236				
NQ version 1or 2	0.00 (0.032)	0.989				
MMSE-NR score	−0.25 (0.003)	**0.011**			−0.17 (0.003)	0.084
APOE ε4-carrier	0.18 (0.012)	0.078	0.03 (0.012)	0.795	−0.01 (0.012)	0.888
*R* ^2^			0.29		0.32	

Hippocampus percentile as the independent variable.

Values in bold are significant. *β*: coefficient; SE: standard error; *R*^2^: explained variance; NQ: NeuroQuant®; MMSE-NR: Mini-Mental State Examination-Norwegian revision.

**Table 9:** Linear regression analysis of annual change in CDR-SB score involving 100 patients with complete data, including APOE **ɛ**4-carrier status.

	Unadjusted		Adjusted model 1		Adjusted model 2	
	β (*SE*)	*p*	β (*SE*)	*p*	β (*SE*)	*p*
Age	0.33 (0.001)	**0.004**	0.17 (0.001)	0.168	0.14 (0.001)	0.245
Sex	−0.42 (0.013)	0.677	−0.05 (0.012)	0.596	−0.04 (0.011)	0.644
Education	−0.16 (0.002)	0.119			−0.06 (0.002)	0.553
Whole brain volume	−0.27 (0.002)	**0.006**	−0.23 (0.002)	0.062	−0.24 (0.002)	**0.043**
MMSE-NR score	−0.25 (0.003)	**0.011**			−0.28 (0.003)	**0.004**
Follow-up time	0.26 (<0.000)	**0.011**	0.31 (0.000)	**0.003**	0.26 (0.00)	**0.007**
MRI NQ before 2015	0.12 (0.013)	0.236				
APOE ε4-carrier	0.18 (0.012)	0.078	0.02 (0.13)	0.853	−0.03 (0.013)	0.799
*R* ^2^			0.20		0.28	

Whole brain volume as the independent variable.

Values in bold are significant. *β*: coefficient; SE: standard error; *R*^2^: explained variance; NQ: NeuroQuant®; MMSE-NR: Mini-Mental State-Examination Norwegian revision.

## Discussion

We found both hippocampus percentile and WBV to be associated with progression, measured by the annual change in CDR-SB scores, in both unadjusted and adjusted analyses. Moreover, hippocampus percentile and WBV were both associated with conversion to a more impaired level; however, when adjusting for baseline cognitive function, these associations were no longer significant. In sub-analyses including *APOE* ɛ4 status as a covariate, hippocampus percentile emerged as a significant predictor of conversion.

In the present study, 15% of the patients converted from SCD to MCI, 6% converted from SCD to dementia, and 25% of the patients converted from MCI to dementia during the follow-up period. The conversion rate for MCI aligns with what has been found in previous studies (50, 51), but is lower compared to findings from a previous Nordic memory clinic study from 2020 (9). Meanwhile, the SCD conversion rate corresponds to what Slot et al. (8) found in a multicenter study on SCD, where 7% converted from SCD to dementia.

We found a significant association between hippocampus percentile and conversion from SCD to MCI or dementia or from MCI to dementia in model 1 but the association lost significance after adjusting for MMSE-NR-score. A possible explanation could be that a lower MMSE-score indicates a more advanced disease stage, which could further be linked to a stage with accelerated cognitive decline (52). In line with this, Mauri et al. (53) found that lower MMSE-score at baseline was a predictor of conversion to dementia in a cohort of patients with MCI. Several studies have shown that hippocampal volume is a valuable predictor of conversion from MCI to AD dementia (15, 54, 55). However, contradictory findings regarding the predictive value of MRI volumetry in patients with SCD have been reported (20, 21). At the same time, Wang et al. (56) concluded in their review from 2020 that the pathological alterations identified by neuroimaging techniques in SCD are parallel to those underlying MCI and AD dementia. Along this line, a recent review of prospective biomarkers in AD, encompassing patients with SCD and MCI, found that decreased volume of the hippocampus predicted clinical progression (54).

In the present study, 80% of the patients who converted to MCI and dementia were diagnosed with clinical AD at follow-up. This may explain the stronger association between hippocampus percentile and conversion than between WBV and conversion. However, 20% of the patients had other types of neuropathology, including vascular pathology, frontotemporal dementia and dementia with Lewy bodies (Figure 1), which may support the fact that MCI and dementia due to other neuropathology can also present with hippocampal atrophy. Conversely, not all AD patients experience hippocampal atrophy (57, 58).

It may be that the association between hippocampal atrophy and WBV and conversion had been strengthened by a longer follow-up time, as the mean follow-up time in this perspective of studying a gradually progressing disease was relatively brief (3.2 years). This lends support to our second outcome measure, specifically progression measured by annual change in CDR-SB, as potentially more suitable considering the constrained follow-up time.

We found that lower hippocampus percentile and WBV at baseline were associated with greater progression of cognitive decline measured by annual change in CDR-SB scores. In line with our finding, Mungas et al. (59) also found an association between hippocampus atrophy at baseline and cognitive decline measured by annual change in CDR scores. Further, a similar association was found in a recent study from Hanseeu et al. (60) following a cohort of clinically healthy older adults over a decade. Moreover, Verfaillie et al. (46) found that widespread cortical thinning in patients with SCD at baseline at a memory clinic was associated with a faster decline in memory over time. The patients were followed for a mean period of 3 years, and a repeated neuropsychological test battery was used to measure progression (46). Progression measured on a continuous scale makes it more sensitive to cognitive and functional decline than when using a categorical scale, as conversion versus non-conversion. This may explain why we found a significant association between the MRI measures and progression but not between the MRI measures and conversion.

Including *APOE* ɛ4 carrier status in the analysis on prediction of conversion strengthened the association between hippocampus percentile and conversion despite a reduced sample size. An analysis on associations between hippocampus percentile and conversion stratified by *APOE* ɛ4 carrier status confirmed an interaction between the *APOE* ɛ4 carrier status and hippocampus percentile. Thus, a significant association between the hippocampus percentile and conversion was found in the *APOE* ɛ4 -negative group but not in the *APOE* ɛ4 -positive group. This might be explained by the fact that *APOE* ɛ4 positivity is a great risk factor for hippocampal atrophy regardless of cognitive disease stage (61) and therefore, in *APOE* ɛ4 negative patients, atrophy will to a higher degree be associated with progression of cognitive decline. Indeed, *APOE* ɛ4 is the strongest genetic risk factor for AD and a risk factor for other neurogenerative diseases (i.e., LBD), as well as a risk factor for age-related cognitive decline (62). A recent expert review has argued that the effect of *APOE* ɛ4 on hippocampus atrophy may be greatest during the transition from MCI to AD (63).

### Strengths and limitations

This study has some limitations. For diagnostic evaluation, we used the current clinical criteria for diagnoses of SCD, MCI, and dementia. Because the diagnoses were made *post hoc* based on patient records, we cannot rule out that some details may have been missing in the reports and could have affected the diagnostic evaluation. Beyond that, it can be challenging to distinguish patients with MCI from patients with an early stage of dementia and younger and/or highly educated patients, as included in the present study, tends to be easier misdiagnosed with the current clinical criteria for the diagnoses of SCD and MCI as they perform better on the cognitive tests (64, 65). These aspects may have caused some patients to be misclassified at baseline or follow-up. Our outcome measures: conversion and annual change in CDR-SB have both their strengths and limitations. The continuous variable: annual change in CDR-SB captures even slight deterioration in cognitive and functional function, whereas the categorical variable; conversion does not capture a decline in cognitive and functional function as long as it does not lead to a change in diagnosis. Another limitation is that specific AD biomarkers were not included in the diagnostic work-up as these examinations were only available in a smaller subset of the patients. However, as the aim was to explore progression in a heterogenous SCD and MCI population, we believe the lack of AD specific biomarkers did not compromise the results, as well as it mimics a naturalistic diagnostic setting. About 50% of the patients which were eligible for inclusion did not have a follow up consultation at the memory clinic. However, analyses comparing the followed-up and the not followed-up did not reveal any differences in baseline characteristics.

We could have included other MRI measures which have shown association with cognitive decline, i.e., white matter hyperintensities (66), however these measures were not available in the beginning years of NQ. As our aim was to explore if an AD-specific measure and a general measure of atrophy could give valuable predictive information to the clinician in patients with early symptoms of cognitive decline, we chose to include only these two measures.

A strength of this study is that all diagnoses were set according to research criteria and the diagnoses, and the CDR-ratings were set by the same researchers and evaluated by two experienced senior clinicians in inconclusive cases. These two aspects ensured consistency and quality in the diagnostic process. Our study included patients referred and followed up in a clinical setting, a drawback with this is a greater vulnerability to missing data, but a strength is the possible increased relevance to clinical practice.

## Conclusion

Overall, our results indicate that automated regional MRI volumetry of the hippocampus and WBV can be useful in predicting further cognitive decline in patients with early cognitive symptoms. The method’s ability to automatically produce volumetric data without the expertise of a neuroradiologist may be a useful tool in identifying patients at risk of developing dementia also at a primary care level.

## Data availability statement

The data analyzed in this study is subject to the following licenses/restrictions: the de-identified dataset generated and analyzed during the current study is not publicly available due to sensitive patient information but is available from the corresponding author on reasonable request. An approval by Regional Committee of Medical Research Ethics of the South-East Norway (email: post@helseforskning.etikkom.no) is required due to legal restrictions. Requests to access these datasets should be directed to rachel.amland@aldringoghelse.no.

## Ethics statement

The patients included in our study are included in the NorCog registry and have given their written informed consent to use their data, including genetic information such as APOE genotype, for research. Our project was approved by the regional Committee of Medical Research Ethics of the South-East Norway (REC South-East number 2019/79).

## Author contributions

RA: Conceptualization, Data curation, Formal analysis, Investigation, Methodology, Project administration, Validation, Visualization, Writing – original draft, Writing – review & editing, Resources. GS: Conceptualization, Funding acquisition, Methodology, Project administration, Supervision, Writing – original draft, Writing – review & editing. AB: Conceptualization, Methodology, Supervision, Writing – original draft, Writing – review & editing. TE: Data curation, Investigation, Writing – review & editing. KE: Conceptualization, Funding acquisition, Writing – review & editing. ABK: Investigation, Writing – review & editing. EO: Investigation, Resources, Writing – review & editing. KP: Conceptualization, Data curation, Formal analysis, Investigation, Methodology, Project administration, Resources, Supervision, Validation, Visualization, Writing – original draft, Writing – review & editing.

## References

[1] World health organization. Dementia fact sheet 2022. Geneva, Switzerland: World Health Organization (2022).

[2] Kivipelto M Mangialasche F Snyder HM Allegri R Andrieu S Arai H. World-wide FINGERS network: a global approach to risk reduction and prevention of dementia. Alzheimers Dement. (2020) 16:1078–94. doi: 10.1002/alz.12123, PMID: 32627328 PMC9527644

[3] Ngandu T Lehtisalo J Solomon A Levälahti E Ahtiluoto S Antikainen R. A 2 year multidomain intervention of diet, exercise, cognitive training, and vascular risk monitoring versus control to prevent cognitive decline in at-risk elderly people (FINGER): a randomised controlled trial. Lancet. (2015) 385:2255–63. doi: 10.1016/S0140-6736(15)60461-5 25771249

[4] Cummings J Zhou Y Lee G Zhong K Fonseca J Cheng F. Alzheimer's disease drug development pipeline: 2023. Alzheimers Dement. (2023) 9:e12385. doi: 10.1002/trc2.12385, PMID: 37251912 PMC10210334

[5] Abdelnour C Gonzalez MC Gibson LL Poston KL Ballard CG Cummings JL. Dementia with Lewy bodies drug therapies in clinical trials: systematic review up to 2022. Neurol Ther. (2023) 12:727–49. doi: 10.1007/s40120-023-00467-8, PMID: 37017910 PMC10195935

[6] Petersen RC. Clinical practice. Mild cognitive impairment. N Engl J Med. (2011) 364:2227–34. doi: 10.1056/NEJMcp0910237 21651394

[7] 2022Alzheimer's disease facts and figures. 2022 Alzheimer's disease facts and figures. Alzheimers Dement. (2022) 18:700–89. doi: 10.1002/alz.12638 35289055

[8] Slot RER Sikkes SAM Berkhof J Brodaty H Buckley R Cavedo E. Subjective cognitive decline and rates of incident Alzheimer's disease and non-Alzheimer's disease dementia. Alzheimers Dement. (2019) 15:465–76. doi: 10.1016/j.jalz.2018.10.003, PMID: 30555032 PMC6465066

[9] Engedal K Barca ML Høgh P Bo Andersen B Winther Dombernowsky N Naik M. The Power of EEG to predict conversion from mild cognitive impairment and subjective cognitive decline to dementia. Dement Geriatr Cogn Disord. (2020) 49:38–47. doi: 10.1159/000508392, PMID: 32610316

[10] Landau SM Harvey D Madison CM Reiman EM Foster NL Aisen PS. Comparing predictors of conversion and decline in mild cognitive impairment. Neurology. (2010) 75:230–8. doi: 10.1212/WNL.0b013e3181e8e8b8, PMID: 20592257 PMC2906178

[11] Wu A Sharrett AR Gottesman RF Power MC Mosley TH Jr Jack CR Jr. Association of Brain Magnetic Resonance Imaging Signs with Cognitive Outcomes in persons with nonimpaired cognition and mild cognitive impairment. JAMA Netw Open. (2019) 2:e193359. doi: 10.1001/jamanetworkopen.2019.3359, PMID: 31074810 PMC6512274

[12] Uchida Y Kan H Sakurai K Oishi K Matsukawa N. Contributions of blood-brain barrier imaging to neurovascular unit pathophysiology of Alzheimer's disease and related dementias. Front Aging Neurosci. (2023) 15:1111448. doi: 10.3389/fnagi.2023.1111448, PMID: 36861122 PMC9969807

[13] Uchida Y Kan H Sakurai K Oishi K Matsukawa N. Quantitative susceptibility mapping as an imaging biomarker for Alzheimer's disease: the expectations and limitations. Front Neurosci. (2022) 16:938092. doi: 10.3389/fnins.2022.938092, PMID: 35992906 PMC9389285

[14] Ruan D Sun L. Amyloid-β PET in Alzheimer's disease: a systematic review and Bayesian meta-analysis. Brain Behav. (2023) 13:e2850. doi: 10.1002/brb3.2850, PMID: 36573329 PMC9847612

[15] Tanpitukpongse TP Mazurowski MA Ikhena J Petrella JR. Predictive utility of marketed volumetric software tools in subjects at risk for Alzheimer disease: do regions outside the hippocampus matter? AJNR Am J Neuroradiol. (2017) 38:546–52. doi: 10.3174/ajnr.A5061, PMID: 28057634 PMC5352470

[16] Frisoni GB Fox NC Jack CR Jr Scheltens P Thompson PM. The clinical use of structural MRI in Alzheimer disease. Nat Rev Neurol. (2010) 6:67–77. doi: 10.1038/nrneurol.2009.215, PMID: 20139996 PMC2938772

[17] McRae-McKee K Evans S Hadjichrysanthou C Wong MM de Wolf F Anderson RM. Combining hippocampal volume metrics to better understand Alzheimer's disease progression in at-risk individuals. Sci Rep. (2019) 9:7499. doi: 10.1038/s41598-019-42632-w, PMID: 31097733 PMC6522521

[18] Leong RLF Lo JC Sim SKY Zheng H Tandi J Zhou J. Longitudinal brain structure and cognitive changes over 8 years in an east Asian cohort. NeuroImage. (2017) 147:852–60. doi: 10.1016/j.neuroimage.2016.10.016, PMID: 27742600

[19] Prosser L Macdougall A Sudre CH Manning EN Malone IB Walsh P. Predicting cognitive decline in older adults using baseline metrics of AD pathologies, cerebrovascular disease, and neurodegeneration. Neurology. (2023) 100:e834–45. doi: 10.1212/WNL.0000000000201572, PMID: 36357185 PMC9984210

[20] Arrondo P Elía-Zudaire Ó Martí-Andrés G Fernández-Seara MA Riverol M. Grey matter changes on brain MRI in subjective cognitive decline: a systematic review. Alzheimers Res Ther. (2022) 14:98. doi: 10.1186/s13195-022-01031-6, PMID: 35869559 PMC9306106

[21] Parker AF Smart CM Scarapicchia V Gawryluk JR. Identification of earlier biomarkers for Alzheimer's disease: a multimodal neuroimaging study of individuals with subjective cognitive decline. J Alzheimers Dis. (2020) 77:1067–76. doi: 10.3233/JAD-200299, PMID: 32804127

[22] Perrotin A La Joie R de La Sayette V Barré L Mézenge F Mutlu J. Subjective cognitive decline in cognitively normal elders from the community or from a memory clinic: differential affective and imaging correlates. Alzheimers Dement. (2017) 13:550–60. doi: 10.1016/j.jalz.2016.08.011, PMID: 27693187

[23] Saykin AJ Wishart HA Rabin LA Santulli RB Flashman LA West JD. Older adults with cognitive complaints show brain atrophy similar to that of amnestic MCI. Neurology. (2006) 67:834–42. doi: 10.1212/01.wnl.0000234032.77541.a2, PMID: 16966547 PMC3488276

[24] Scheef L Spottke A Daerr M Joe A Striepens N Kölsch H. Glucose metabolism, gray matter structure, and memory decline in subjective memory impairment. Neurology. (2012) 79:1332–9. doi: 10.1212/WNL.0b013e31826c1a8d, PMID: 22914828

[25] Caillaud M Hudon C Boller B Brambati S Duchesne S Lorrain D. Evidence of a relation between hippocampal volume, white matter Hyperintensities, and cognition in subjective cognitive decline and mild cognitive impairment. J Gerontol B Psychol Sci Soc Sci. (2020) 75:1382–92. doi: 10.1093/geronb/gbz120, PMID: 31758692 PMC7424270

[26] Selnes P Fjell AM Gjerstad L Bjørnerud A Wallin A Due-Tønnessen P. White matter imaging changes in subjective and mild cognitive impairment. Alzheimers Dement. (2012) 8:S112–21. doi: 10.1016/j.jalz.2011.07.001, PMID: 23021621

[27] Persson K Barca ML Cavallin L Brækhus A Knapskog AB Selbæk G. Comparison of automated volumetry of the hippocampus using NeuroQuant® and visual assessment of the medial temporal lobe in Alzheimer's disease. Acta Radiol. (2018) 59:997–1001. doi: 10.1177/0284185117743778, PMID: 29172642

[28] Pini L Wennberg AM. Structural imaging outcomes in subjective cognitive decline: community vs. clinical-based samples. Exp Gerontol. (2021) 145:111216. doi: 10.1016/j.exger.2020.111216, PMID: 33340685

[29] Ross DE Ochs AL Tate DF Tokac U Seabaugh J Abildskov TJ. High correlations between MRI brain volume measurements based on NeuroQuant(®) and FreeSurfer. Psychiatry Res Neuroimaging. (2018) 278:69–76. doi: 10.1016/j.pscychresns.2018.05.007, PMID: 29880256

[30] Persson K Barca ML Edwin TH Cavallin-Eklund L Tangen GG Rhodius-Meester HFM. Regional MRI volumetry using NeuroQuant versus visual rating scales in patients with cognitive impairment and dementia. Brain Behav. (2024) 14:e3397. doi: 10.1002/brb3.3397, PMID: 38600026 PMC10839122

[31] Yim Y Lee JY Oh SW Chung MS Park JE Moon Y. Comparison of automated brain volume measures by NeuroQuant vs. Freesurfer in patients with mild cognitive impairment: effect of slice thickness. Yonsei Med J. (2021) 62:255–61. doi: 10.3349/ymj.2021.62.3.255, PMID: 33635016 PMC7934099

[32] Song H Lee SA Jo SW Chang SK Lim Y Yoo YS. Agreement and reliability between clinically available software programs in measuring volumes and normative percentiles of segmented brain regions. Korean J Radiol. (2022) 23:959–75. doi: 10.3348/kjr.2022.0067, PMID: 36175000 PMC9523231

[33] Raji CA Benzinger TLS. The value of neuroimaging in dementia diagnosis. Continuum. (2022) 28:800–21. doi: 10.1212/CON.0000000000001133, PMID: 35678403 PMC9993425

[34] Medbøen IT Persson K Nåvik M Totland TH Bergh S Treviño CS. Cohort profile: the Norwegian registry of persons assessed for cognitive symptoms (NorCog) - a national research and quality registry with a biomaterial collection. BMJ Open. (2022) 12:e058810. doi: 10.1136/bmjopen-2021-058810, PMID: 36448543 PMC9462106

[35] Folstein MF Folstein SE McHugh PR. "mini-mental state". A practical method for grading the cognitive state of patients for the clinician. J Psychiatr Res. (1975) 12:189–98. doi: 10.1016/0022-3956(75)90026-6 1202204

[36] Hughes CP Berg L Danziger WL Coben LA Martin RL. A new clinical scale for the staging of dementia. Br J Psychiatry. (1982) 140:566–72. doi: 10.1192/bjp.140.6.566 7104545

[37] O'Bryant SE Waring SC Cullum CM Hall J Lacritz L Massman PJ. Staging dementia using clinical dementia rating scale sum of boxes scores: a Texas Alzheimer's research consortium study. Arch Neurol. (2008) 65:1091–5. doi: 10.1001/archneur.65.8.1091 18695059 PMC3409562

[38] Jessen F Amariglio RE van Boxtel M Breteler M Ceccaldi M Chételat G. A conceptual framework for research on subjective cognitive decline in preclinical Alzheimer's disease. Alzheimers Dement. (2014) 10:844–52. doi: 10.1016/j.jalz.2014.01.001, PMID: 24798886 PMC4317324

[39] Albert MS DeKosky ST Dickson D Dubois B Feldman HH Fox NC. The diagnosis of mild cognitive impairment due to Alzheimer's disease: recommendations from the National Institute on Aging-Alzheimer's Association workgroups on diagnostic guidelines for Alzheimer's disease. Alzheimers Dement. (2011) 7:270–9. doi: 10.1016/j.jalz.2011.03.008, PMID: 21514249 PMC3312027

[40] American Psychiatric Association. Diagnostic and statistical manual of mental disorders. *5th edn.* Washington, DC: American Psychiatric Publishing. (2013). doi: 10.1176/appi.books.9780890425596

[41] McKhann GM Knopman DS Chertkow H Hyman BT Jack CR Jr Kawas CH. The diagnosis of dementia due to Alzheimer's disease: recommendations from the National Institute on Aging-Alzheimer's Publishing workgroups on diagnostic guidelines for Alzheimer's disease. Alzheimers Dement. (2011) 7:263–9. doi: 10.1016/j.jalz.2011.03.005, PMID: 21514250 PMC3312024

[42] Sachdev P Kalaria R O'Brien J Skoog I Alladi S Black SE. Diagnostic criteria for vascular cognitive disorders: a VASCOG statement. Alzheimer Dis Assoc Disord. (2014) 28:206–18. doi: 10.1097/WAD.0000000000000034, PMID: 24632990 PMC4139434

[43] McKeith IG Boeve BF Dickson DW Halliday G Taylor JP Weintraub D. Diagnosis and management of dementia with Lewy bodies: fourth consensus report of the DLB consortium. Neurology. (2017) 89:88–100. doi: 10.1212/WNL.0000000000004058, PMID: 28592453 PMC5496518

[44] Rascovsky K Hodges JR Knopman D Mendez MF Kramer JH Neuhaus J. Sensitivity of revised diagnostic criteria for the behavioural variant of frontotemporal dementia. Brain. (2011) 134:2456–77. doi: 10.1093/brain/awr179, PMID: 21810890 PMC3170532

[45] Gorno-Tempini ML Hillis AE Weintraub S Kertesz A Mendez M Cappa SF. Classification of primary progressive aphasia and its variants. Neurology. (2011) 76:1006–14. doi: 10.1212/WNL.0b013e31821103e6 21325651 PMC3059138

[46] Verfaillie SCJ Slot RE Tijms BM Bouwman F Benedictus MR Overbeek JM. Thinner cortex in patients with subjective cognitive decline is associated with steeper decline of memory. Neurobiol Aging. (2018) 61:238–44. doi: 10.1016/j.neurobiolaging.2017.09.009, PMID: 29029762

[47] Hall JM Lewis SJG. Neural correlates of cognitive impairment in Parkinson's disease: a review of structural MRI findings. Int Rev Neurobiol. (2019) 144:1–28. doi: 10.1016/bs.irn.2018.09.009, PMID: 30638452

[48] Knopman DS Mosley TH Catellier DJ Coker LH. Fourteen-year longitudinal study of vascular risk factors, APOE genotype, and cognition: the ARIC MRI study. Alzheimers Dement. (2009) 5:207–14. doi: 10.1016/j.jalz.2009.01.027, PMID: 19362884

[49] Donix M Burggren AC Scharf M Marschner K Suthana NA Siddarth P. APOE associated hemispheric asymmetry of entorhinal cortical thickness in aging and Alzheimer's disease. Psychiatry Res. (2013) 214:212–20. doi: 10.1016/j.pscychresns.2013.09.006, PMID: 24080518 PMC3851589

[50] Bruscoli M Lovestone S. Is MCI really just early dementia? A systematic review of conversion studies. Int Psychogeriatr. (2004) 16:129–40. doi: 10.1017/S1041610204000092, PMID: 15318760

[51] Mitchell AJ Shiri-Feshki M. Rate of progression of mild cognitive impairment to dementia – meta‐analysis of 41 robust inception cohort studies. Acta Psychiatr Scand. (2009) 119:252–65. doi: 10.1111/j.1600-0447.2008.01326.x 19236314

[52] Edwin TH Strand BH Persson K Engedal K Selbæk G Knapskog AB. Trajectories and risk factors of dementia progression: a memory clinic cohort followed up to 3 years from diagnosis. Int Psychogeriatr. (2021) 33:779–89. doi: 10.1017/S1041610220003270, PMID: 33213607

[53] Mauri M Sinforiani E Zucchella C Cuzzoni MG Bono G. Progression to dementia in a population with amnestic mild cognitive impairment: clinical variables associated with conversion. Funct Neurol. (2012) 27:49–54. PMID: 22687167 PMC3812753

[54] Li RX Ma YH Tan L Yu JT. Prospective biomarkers of Alzheimer's disease: a systematic review and meta-analysis. Ageing Res Rev. (2022) 81:101699. doi: 10.1016/j.arr.2022.101699, PMID: 35905816

[55] Brueggen K Dyrba M Barkhof F Hausner L Filippi M Nestor PJ. Basal forebrain and hippocampus as predictors of conversion to Alzheimer's disease in patients with mild cognitive impairment - a Multicenter DTI and Volumetry study. J Alzheimers Dis. (2015) 48:197–204. doi: 10.3233/JAD-150063, PMID: 26401940

[56] Wang X Huang W Su L Xing Y Jessen F Sun Y. Neuroimaging advances regarding subjective cognitive decline in preclinical Alzheimer's disease. Mol Neurodegener. (2020) 15:55. doi: 10.1186/s13024-020-00395-3, PMID: 32962744 PMC7507636

[57] Pini L Pievani M Bocchetta M Altomare D Bosco P Cavedo E. Brain atrophy in Alzheimer's disease and aging. Ageing Res Rev. (2016) 30:25–48. doi: 10.1016/j.arr.2016.01.002 26827786

[58] Persson K Edwin TH Knapskog AB Tangen GG Selbæk G Engedal K. Hippocampal atrophy subtypes of Alzheimer's disease using automatic MRI in a memory clinic cohort: clinical implications. Dement Geriatr Cogn Disord. (2022) 51:80–9. doi: 10.1159/000522382, PMID: 35344967

[59] Mungas D Harvey D Reed BR Jagust WJ DeCarli C Beckett L. Longitudinal volumetric MRI change and rate of cognitive decline. Neurology. (2005) 65:565–71. doi: 10.1212/01.wnl.0000172913.88973.0d, PMID: 16116117 PMC1820871

[60] Hanseeuw BJ Jacobs HI Schultz AP Buckley RF Farrell ME Guehl NJ. Association of pathological and volumetric biomarker changes with cognitive decline in clinically normal adults: Harvard aging brain study. Neurology. (2023) 2533–2544. doi: 10.1212/WNL.0000000000207962 PMC10791053 37968130

[61] Shi J Leporé N Gutman BA Thompson PM Baxter LC Caselli RJ. Genetic influence of apolipoprotein E4 genotype on hippocampal morphometry: an N = 725 surface-based Alzheimer's disease neuroimaging initiative study. Hum Brain Mapp. (2014) 35:3903–18. doi: 10.1002/hbm.22447, PMID: 24453132 PMC4269525

[62] Martens YA Zhao N Liu CC Kanekiyo T Yang AJ Goate AM. ApoE Cascade hypothesis in the pathogenesis of Alzheimer's disease and related dementias. Neuron. (2022) 110:1304–17. doi: 10.1016/j.neuron.2022.03.004, PMID: 35298921 PMC9035117

[63] Saeed U Desmarais P Masellis M. The APOE ε4 variant and hippocampal atrophy in Alzheimer's disease and Lewy body dementia: a systematic review of magnetic resonance imaging studies and therapeutic relevance. Expert Rev Neurother. (2021) 21:851–70. doi: 10.1080/14737175.2021.1956904, PMID: 34311631

[64] Engedal K Benth J Gjøra L Skjellegrind HK Nåvik M Selbæk G. Normative scores on the Norwegian version of the mini-mental state examination. J Alzheimers Dis. (2023) 92:831–42. doi: 10.3233/JAD-221068, PMID: 36847004

[65] Wagle J Selbæk G Benth J Gjøra L Rønqvist TK Bekkhus-Wetterberg P. The CERAD word list memory test: normative data based on a Norwegian population-based sample of healthy older adults 70 years and above. HUNT Study J Alzheimers Dis. (2023) 91:321–43. doi: 10.3233/JAD-220672, PMID: 36404547

[66] Yamanaka T Uchida Y Sakurai K Kato D Mizuno M Sato T. Anatomical links between white matter Hyperintensity and medial temporal atrophy reveal impairment of executive functions. Aging Dis. (2019) 10:711–8. doi: 10.14336/AD.2018.0929, PMID: 31440378 PMC6675535

