# Supplementary material for: Clinically feasible automated MRI volumetry of the brain as a prognostic marker in subjective and mild cognitive impairment
Source: Front Neurol. 2024 Jul 1;15:1425502. doi: 10.3389/fneur.2024.1425502 (PMC11248186; doi:10.3389/fneur.2024.1425502)
Supplement: Supplementary file 1 [file Data_Sheet_1.docx]

# ***Supplementary Material***

**Clinically feasible automated MRI volumetry of the brain as a prognostic marker in subjective and mild cognitive impairment**

Rachel Amland^*^, Geir Selbæk, Anne Brækhus, Trine H. Edwin, Knut Engedal, Anne-Brita Knapskog, Ellen Regine Olsrud, Karin Persson

Corresponding Author: [rachel.amland@aldringoghelse.no](mailto:rachel.amland@aldringoghelse.no) (RA)

1. **Supplementary Tables**

**Supplementary Table 1**. Fully adjusted logistic regression analyses with converters (1) and non-converters (0) as dependent variable involving 141 patients with complete data. Hippocampus percentile and whole brain as the independent variables in two separate analyses (column 1 and column 2) (DOCX)

**Supplementary Table 2**. Fully adjusted linear regression analyses of annual change in CDR-SB score involving 117 patients with complete data. Hippocampus and whole brain as independent variables, in two separate analyses (column 1 and column 2) (DOCX)

**Supplementary Table 3.** Characteristics of the SCD patients (DOCX)

**Supplementary Table 4**. Logistic regression with patients converting to AD-MCI or AD-dementia (1) versus non-AD-converters (0) as dependent variable involving 141 patients with complete data. Hippocampus percentile as the independent variable. (DOCX)

# Supplementary tables

***S1 Table: Fully adjusted logistic regression analyses with converters (1) and non-converters (0) as dependent* involving 141 patients with complete data. *Hippocampus percentile and whole brain as the independent variables in two separate analyses (column 1 and column 2)***

|  | Fully adjusted model with hippocampus percentile as independent variable | | Fully adjusted model with whole brain volume as independent variable. | |
| --- | --- | --- | --- | --- |
|  | OR (95% CI) | *p* | OR (95% CI) | *p* |
| Age | 1.05 (0.1.0–1.11) | 0.074 | 1.05 (0.98–1.13) | 0.141 |
| Sex | 0.48 (0.20–1.17) | 0.106 | 0.548 (0.23–1.29) | 0.167 |
| Education | 0.98 (0.86–1.12) | 0.708 | 0.988 (0.87-1.12) | 0.845 |
| Hippocampus percentile | 0.98 (0.97–0.99) | 0.053 |  |  |
| Whole brain volume |  |  | 0.95 (0.80-1.12) | 0.506 |
| Follow-up time | 1.05 (1.02–1.03) | **0.003** | 1.05 (1.02–1.08) | **0.002** |
| MRI scanner before 2015 | 0.44 (0.15–1.28) | 0.132 | 0.543 (0.175-1.687) | 0.291 |
| NQ version 1 or 2 (only relevant for the hippocampus NQ measure) | 0.94 (0.24-4.61) | 0.944 |  |  |
| MMSE-NR score | 0.85 (0.70–1.03) | 0.104 | 0.82 (0.68-1.00) | **0.044** |
| *R*^2^ | 0.26 | | 0.23 | |
| *Values in bold are significant, OR: odds ratio; CI: confidence interval; R2: explained variance, NQ: NeuroQuant®; MMSE-NR: Mini-Mental State Examination-Norwegian revision.* | | | | |

***S2 Table: Fully adjusted linear regression analyses of annual change in CDR-SB score involving 117 patients with complete data. Hippocampus and whole brain as independent variables, in two separate analyses (column 1 and column 2)***

|  | Fully adjusted model with hippocampus percentile as independent variable | | Fully adjusted model with whole brain volume as independent variable. | |
| --- | --- | --- | --- | --- |
|  | β (SE) | *p* | β (SE) | *p* |
| Age | 0.17 (0.00) | 0.070 | 0.096 (0.00) | 0.402 |
| Sex | -0.11 (0.11) | 0.212 | -0.07 (0.11) | 0.432 |
| Education | -0.07 (0.00) | 0.438 | -0.05 (0.00) | 0.535 |
| Hippocampus percentile | -0.25 (0.00) | **0.013** |  |  |
| Whole brain volume |  |  | -0,26 (0.00) | **0.035** |
| Follow-up time | 0.32 (0.00) | **0.002** | 0.36 (0.00) | **>0.001** |
| MRI scanner before 2015 | -0.13 (0.13) | 0.219 | -0.03 (0.14) | 0.742 |
| NQ version 1 or 2 (only relevant for the hippocampus NQ measure) | 0.09 (0.02) | 0.298 |  |  |
| MMSE-NR score | -0.21 (0.00) | **0.024** | -0,27 (0.00) | **0.004** |
| *R*^2^ | 0.22 | | 0.20 | |
| *Values in bold are significant. β: coefficient; SE: standard error; R^2^: explained variance; NQ: NeuroQuant®; MMSE-NR: Mini-Mental State Examination-Norwegian revision.* | | | | |

***S3 Table. Characteristics of the SCD patients***

|  | SCD converters  n 10 | SCD non-converters  n=36 | pᶧ |
| --- | --- | --- | --- |
| Age, years | 70.2 (5.4) | 60.3 (9.5) | **0.003** |
| Female, *n* (%) | 18 (50.0) | 6 (60.0) | 0.575 |
| Education, years | 15.4 (4.0) | 14.6 (3.6) | 0.555 |
| MMSE-NR score at baseline | 29.3 (1.0) | 29.1 (1.0) | 0.521 |
| CDR-SB score at baseline | 0.4 (0.5) | 0.3 (0.4) | 0.673 |
| Annual change in CDR-SB score* | 0.04 (0.7) | 0.00(0.3) | **0.021** |
| APOE ɛ4 carriers, *n* (%)** | 5 (71.4.) | 9 (30.0) | **0.042** |
| Hippocampus percentile | 35.0 (30.7) | 63.0 (32.9) | **0.010** |
| Whole brain volume | 72.6 (3.3) | 75.3 (3.2) | **0.025** |
| Follow-up time (in years) | 3.6 (1.6) | 2.2 (0.9) | **0.003** |
| *All continuous variables are expressed as mean (SD). Bold values highlight significant differences (p < 0.05 two-tailed); MMSE-NR: Mini-Mental State Examination-Norwegian revision; CDR-SB: Clinical Dementia Rating Scale Sum of Boxes;* ᶧ*Student’s t test/χ2 test.  *n 40, ** n 37* | | | |

**S4 Table: Logistic regression with patients converting to AD-MCI or AD-dementia (1) versus non-AD-converters (0) as dependent variable involving 141 patients with complete data. Hippocampus percentile as the independent variable**

|  | Unadjusted | | Adjusted model 2 | |
| --- | --- | --- | --- | --- |
|  | OR (95% CI) | p | OR (95% CI) | p |
| Age | 1.10 (1.04-1.16) | **<0.001** | 1.07 (1.01-1.14) | **0.028** |
| Sex | 0.42 (0.22-1.13) | 0.094 | 0.34 (0.13-0.91) | **0.032** |
| Education | 0.99 (0.88-1.12) | 0.850 |  |  |
| Hippocampus percentile | 0.98 (0.96-0.99) | **0.003** | 0.98 (0.97-1.00) | 0.069 |
| Follow up time | 1.02 (1.00-1.05) | **0.039** | 1.04 (1.01-1.07) | **0.006** |
| MRI NQ before 2015 | 1.15 (0.51-2.60) | 0.745 |  |  |
| NQ version 1 or 2 | 0.88 (0.38-2.00) | 0.752 |  |  |
| MMSE-NR score | 0.80 (0.67-0.95) | **0.012** | 0.81 (0.66-0.99) | **0.036** |
| R2 |  |  |  | 0.32 |
| Values in bold are significant, OR: Odds ratio; CI: Confidence interval, R2: explained variance, NQ: NeuroQuant®; MMSE-NR: Mini-Mental State-Examination Norwegian revision. | | | | |
